# Complete Genome Sequence of a Sequence Type 4846 Streptococcus pneumoniae Serotype 12F Strain Isolated from a Meningitis Case in Japan

**DOI:** 10.1128/MRA.01632-18

**Published:** 2019-03-14

**Authors:** Bin Chang, Masatomo Morita, Ken-ichi Lee, Makoto Ohnishi

**Affiliations:** a Department of Bacteriology I, National Institute of Infectious Diseases, Tokyo, Japan; University of Rochester School of Medicine and Dentistry

## Abstract

Streptococcus pneumoniae serotype 12F rarely colonizes the nasopharynx but commonly causes invasive pneumococcal disease. Here, we report the complete genome sequence of a sequence type 4846 (ST4846) S. pneumoniae serotype 12F strain isolated from a cluster of invasive pneumococcal disease patients in Japan.

## ANNOUNCEMENT

Streptococcus pneumoniae is a globally prominent human pathogen, and it is the main cause of otitis media, pneumonia, bacteremia, and meningitis. Colonization in the nasopharynx is a prerequisite for the onset of pneumococcal diseases (1, 2). S. pneumoniae serotype 12F generally causes invasive pneumococcal disease (IPD); however, it is rarely present in colonizing isolates (3, 4). Of note, several outbreak infections caused by serotype 12F S. pneumoniae have been reported (5, 6). In 2016, we experienced a community cluster IPD episode caused by serotype 12F S. pneumoniae—9 episodes occurred from March to May 2016 in Tsuruoka City, Japan (7). The isolates were confirmed to be S. pneumoniae by determination of the S. pneumoniae-specific *lytA* gene using the method described by Llull et al. (8).

To clarify the characteristics of the 12F S. pneumoniae strains causing the cluster IPD infection, we determined the whole genome of strain ASP0581, which was isolated from the first case. The patient was an 89-year-old female, and the onset of disease was on 28 March 2016. She was diagnosed with meningitis and arthritis of the right knee. After treatment with antibiotics, she recovered and was discharged from the hospital.

The S. pneumoniae strain ASP0581 was isolated from spinal fluid cultured in a blood bottle and plated on Columbia agar with 5% sheep blood (Becton, Dickinson and Company Japan, Tokyo, Japan) overnight at 37°C with 5% CO_2_. Genomic DNA was purified from the strain using the Wizard genomic DNA purification kit (Promega, Madison, WI). Short-read sequences of the strain were obtained on a MiSeq instrument (Illumina, San Diego, CA). The genomic DNA libraries were prepared using a Nextera XT DNA sample preparation kit (Illumina). The pooled libraries were subjected to multiplexed paired-end sequencing (300-mer × 2). Additionally, ASP0581 was also sequenced on a PacBio RS II instrument (Pacific Biosciences, Menlo Park, CA) at TaKaRa Bio, Inc. (Shiga, Japan). The genomic DNA was fragmented prior to PacBio RS II sequencing using the Covaris g-Tube device (Woburn, MA), in accordance with the manufacturer’s instructions. PacBio RS II sequencing runs were performed using the PacBio SMRTbell template prep kit 1.0 and polymerase binding kit P6 after size selection using BluePippin (Sage Science, Beverly, MA) with a cutoff value of 15 kb.

The complete genome of strain ASP0581 was obtained by using both PacBio RS II and Illumina reads. PacBio RS II reads were assembled using the Hierarchical Genome Assembly Process (HGAP) version 3 in SMRT Analysis software (Pacific Biosciences) and Canu software version 1.5 (9). The minimum seed length for HGAP assembly was 6,000. The total length and total number of PacBio RS II reads were 1,018,169,410 bases and 76,206 reads, respectively. Illumina short reads were mapped to the contigs obtained from the HGAP assembly using CLC Genomic Workbench version 8.5.1 (Qiagen, Venlo, Netherlands). The length and number of Illumina reads were 619,654,081 bases and 2,811,564 reads, respectively, in total. Contigs from the two assemblies were compared, confirmed by Sanger sequencing, and manually curated.

The ASP0581 genome sequence was confirmed by Sanger sequencing to be closed. Annotation was performed using DDBJ Fast Annotation and Submission Tool (10).

The complete genome sequence of ASP0581 was 2,155,932 bp, with 39.5% G+C content. We observed 2,161 protein-coding regions, 4 rRNA operons, and 58 tRNA genes. The genomic sequence of the strain was queried on the pneumococcal MLST website (https://pubmlst.org/spneumoniae/). Allelic numbers of seven housekeeping genes (*aroE*, *gdh*, *gki*, *recP*, *spi*, *xpt*, *ddl*) and a sequence type (ST) were assigned. The ST of ASP0581 was determined to be ST4846.

These data might help comparisons between the 12F S. pneumoniae strains that cause infections in different regions of Japan and with 12F strains isolated from countries and regions other than Japan.

### Data availability.

The whole-genome sequence of Streptococcus pneumoniae serotype 12F strain ASP0581 was submitted to DDBJ/ENA/GenBank under the accession number AP019192. Primary data were deposited in the NCBI primary data archive, SRA, under the reference number DRR121434. The version described in this paper is the first version.

## References

[1] Bogaert D, de Groot R, Hermans PWM 2004 *Streptococcus pneumoniae* colonisation: the key to pneumococcal disease. Lancet Infect Dis 4:144–154. doi:10.1016/S1473-3099(04)00938-7. 14998500

[2] Simell B, Auranen K, Käyhty H, Goldblatt D, Dagan R, O’Brien KL, Pneumococcal Carriage Group 2012 The fundamental link between pneumococcal carriage and disease. Expert Rev Vaccines 11:841–855. doi:10.1586/erv.12.53. 22913260

[3] Sleeman KL, Griffiths D, Shackley F, Diggle L, Gupta S, Maiden MC, Moxon ER, Crook DW, Peto TEA 2006 Capsular serotype-specific attack rates and duration of carriage of *Streptococcus pneumoniae* in a population of children. J Infect DIS 194:682–688. doi:10.1086/505710. 16897668

[4] Sandgren A, Sjöström K, Olsson Liljequist B, Christensson B, Samuelsson A, Kronvall G, Henriques Normark B 2004 Effect of clonal and serotype-specific properties on the invasive capacity of *Streptococcus pneumoniae*. J Infect Dis 189:785–796. doi:10.1086/381686. 14976594

[5] Cherian T, Steinhoff MC, Harrison LH, Rohn D, McDougal LK, Dick J 1994 A cluster of invasive pneumococcal disease in young children in child care. JAMA 271:695–697. doi:10.1001/jama.1994.03510330073037. 8309033

[6] Zulz T, Wenger JD, Rudolph K, Robinson DA, Rakov AV, Bruden D, Singleton RJ, Bruce MG, Hennessy TW 2013 Molecular characterization of *Streptococcus pneumoniae* serotype 12F isolates associated with rural community outbreaks in Alaska. J Clin Microbiol 51:1402–1407. doi:10.1128/JCM.02880-12. 23408692 PMC3647894

[7] Ikuse T, Habuka R, Wakamatsu Y, Nakajima T, Saitoh N, Yoshida H, Chang B, Morita M, Ohnishi M, Oishi K, Saitoh A 2018 Local outbreak of *Streptococcus pneumoniae* serotype 12F caused high morbidity and mortality among children and adults. Epidemiol Infect 146:1793–1796. doi:10.1017/S0950268818002133. 30070189 PMC9506687

[8] Llull D, López R, García E 2006 Characteristic signatures of the *lytA* gene provide a basis for rapid and reliable diagnosis of *Streptococcus pneumoniae* infections. J Clin Microbiol 44:1250–1256. doi:10.1128/JCM.44.4.1250-1256.2006. 16597847 PMC1448622

[9] Koren S, Walenz BP, Berlin K, Miller JR, Bergman NH, Phillippy AM 2017 Canu: scalable and accurate long-read assembly via adaptive *k*-mer weighting and repeat separation. Genome Res 27:722–736. doi:10.1101/gr.215087.116. 28298431 PMC5411767

[10] Tanizawa Y, Fujisawa T, Nakamura Y 2018 DFAST: a flexible prokaryotic genome annotation pipeline for faster genome publication. Bioinformatics 34:1037–1039. doi:10.1093/bioinformatics/btx713. 29106469 PMC5860143

